# Evaluation of adding autologous platelet-rich plasma to embryo transfer media on implantation outcomes: An RCT

**DOI:** 10.18502/ijrm.v23i4.18785

**Published:** 2025-06-10

**Authors:** Marzie Sanuie Farimani, Ashraf Aleyasin, Ashraf Moini, Iraj Amiri, Jalal Poorolajal, Fahime Salari Shahrbabaki

**Affiliations:** ^1^Fertility and Infertility Research Center, Hamadan University of Medical Sciences, Hamadan, Iran.; ^2^Department of Infertility, Shariati Hospital, Tehran University of Medical Sciences, Tehran, Iran.; ^3^Department of Infertility, Arash Hospital, Tehran University of Medical Sciences, Tehran, Iran.; ^4^Department of Epidemiology, School of Public Health, Hamadan University of Medical Sciences, Hamadan, Iran.

**Keywords:** Embryo implantation, Platelet-rich plasma, Embryo transfer.

## Abstract

**Background:**

Several recent studies have shown the beneficial effect of platelet-rich plasma (PRP) in improving endometrial function in cases with repeated implantation failure and thin endometrium. However, the mechanism of this effect is unclear.

**Objective:**

To investigate the direct effect of PRP on the implantation process during embryo transfer (ET) in cases without an obvious history of abnormality on implantation.

**Materials and Methods:**

In this randomized clinical trial study, 55 infertile women (20–40 yr) who were candidates for intracytoplasmic sperm injection referred to the Infertility Center of Fatemiyeh hospital in Hamadan, Iran between September 2022 and 2023were randomly divided into intervention and control groups. In the intervention group, PRP was added to the ET medium, while in the control group, a routine ET medium was used. After ET, chemical and clinical pregnancy were measured.

**Results:**

In the intervention and control groups beta human chorionic gonadotropin positivity 14 days after transfer was 33.3% and 39.3%, consecutively (p = 0.646). On day 21 post-transfer, a gestational sac was observed in 33.3% and 35.7% of cases (p = 0.646). By 28 days after transfer fetal heart activity was detected in 33.3% and 35.7% of cases (p = 0.631).

**Conclusion:**

The addition of autologous PRP at the time of ET does not have a direct positive effect on embryo implantation, and the observation of positive effects in previous studies may be indirectly on the uterine microenvironment, which requires time.

## 1. Introduction

Infertility is one of the most important life crises that leads to various personal, family, psychological, economic, and social problems in couples and even lead to divorce (1, 2). Over the past few decades, relatively successful treatment approaches have been developed to treat almost all causes of infertility (3).

The blastocyst is located in the uterine cavity and then goes through 3 stages for implantation, including apposition, adhesion, and invasion stages (4). The final stage is inhibited by specific genetic signals and codes. The blastocyst secretes interleukin 1-α and 1-β, and these cytokines are likely to affect the endometrium directly. Also, embryos secrete human chorionic gonadotropin (hCG), which can affect the acceptability capacity of endometrial (5).

Platelet-rich plasma (PRP) is the plasma volume whose platelet count is higher than normal. PRP contains significant amounts of growth factors, chemokines, cytokines, and active metabolites that act through paracrine on various cells such as myocytes, mesenchymal cells, chondrocytes, osteoblasts, and fibroblasts (6). PRP is inexpensive and easy to obtain, and is a rich growth factor in which platelet growth factors are derived from a person's blood and are therefore nontoxic and nonallergic (7). PRP is used in various medical conditions as adjuvant therapy. It has been used successfully in various fields, such as orthopedics, wound healing and regeneration, and ophthalmology (8–10).

A recent study has shown that this product can positively affect endometrial thickness, and thus increase fertility (11). Platelets release many growth factors that can stimulate the growth of bovine embryos (12). Platelet-derived vascular endothelial growth factor plays an important role in embryo implantation success. PRP enriches the uterine environment with the necessary factors for embryo growth. Many of these factors effectively communicate between the mother and embryo during endometrial decidualization implantation, placental formation, and embryogenesis. Also, PRP injection before embryo transfer (ET) may increase the success of implantation (13). Recently, studies have been performed with this hypothesis that the addition of PRP to sperm may affect the signals that the embryo sends to the mother (14, 15). Despite many discoveries in this field, part of platelet growth factor that is effective in the implantation process is still unclear.

Considering the importance of infertility and also in line with the country's population policies and the possible role of PRP in increasing fertility and to find the exact effect of PRP on which implantation stage, we decided to evaluate the effect of PRP addition to the transfer medium in women under intracytoplasmic sperm injection (ICSI) cycles.

## 2. Materials and Methods

### Study design and participants

In this randomized clinical trial, 56 infertile women aged 20–40 yr, referred to the in vitro fertilization Center of Fatemiyeh hospital in Hamadan, Iran, between September 2022 and 2023, were enrolled.

The inclusion criteria were having at least 2 good-quality embryos available for transfer and a healthy uterine cavity. The exclusion criteria included candidates requiring sperm or oocyte donation, individuals with a previous history of ET, and those with endocrine disorders such as hyperthyroidism, hypothyroidism, hyperprolactinemia, or diabetes.

Eligible women, all experiencing male-factor infertility, were randomly assigned to 2 groups: an intervention group receiving autologous PRP added to the ET medium, and a control group without PRP.

The primary outcomes were: 1) chemical pregnancy, assessed 14 days post-intervention via laboratory testing; 2) clinical pregnancy, assessed 21 days post-intervention using sonography; and 3) detection of fetal heart activity, measured 28 days post-intervention, also via sonography. The secondary outcome was the occurrence of abortion, determined by patient history at 28 days post-intervention.

Based on the estimated effect size, a sample size of 28 participants per group (n = 56) was calculated to achieve 99% confidence (α = 0.01) and 98% power (β = 0.02).


$$n=\frac{2{\left[\left(Z_{1-\frac{\alpha }{2}}\right)+Z_{1-\beta })\right]}^{2}\times \bar{P}\left(1-\bar{P}\right)}{{\left(P_{1}-P_{2}\right)}^{2}}=\frac{2{\left(3.291+1.96\right)}^{2}\times 0.50\left(1-0.50\right)}{{\left(0.50\right)}^{2}}=56$$

The primary outcomes include chemical pregnancy, assessed 14 days after the intervention through laboratory testing; clinical pregnancy, evaluated 21 days after the intervention via sonography; and detection of fetal heart activity, measured 28 days after the intervention, also using sonography. The secondary outcome is the occurrence of abortion, which was documented based on history at 28 ${}^{\text{th}}$ day post intervention.

### Collection and evaluation of seminal plasma properties

The semen samples of males were collected after 2–7 days of abstinence and were prepared using gradient solutions of 80% and 40% and sperm medium (Ham's F10 medium, Sigma-Alderich, USA). Semen characteristics (count, motility, morphology) were analyzed using an optical microscope based on World Health Organization (2021) standards using computer system sperm analysis (CASA) Video Test-Sperm3.2, Video Test Limited Co. Russa (16).

### Ovarian stimulation protocol and oocyte collection

Pituitary suppression was achieved using the gonadotropin-releasing hormone antagonist Ganirelix (Merck Sharp & Dohme, Rome, Italy), while ovarian stimulation was conducted with recombinant follicle-stimulating hormone (rFSH), specifically Puregon (Merck Sharp & Dohme, Rome, Italy) or Gonal-F (Merck, Rome, Italy). Dosages during treatment were tailored individually based on individual characteristics and their response to gonadotropins. Ovulation was triggered with 250–500 IU of recombinant hCG (Ovitrelle, Merck, Rome, Italy), administered 36 hr prior to oocyte retrieval once follicles measuring ≥ 17–18 mm in diameter were detected.

### ICSI

Cumulus-oocyte complexes were incubated in a fertilization medium (Sequential Fert, Origio, Måløv, Denmark). Cumulus cells were eliminated by brief exposure to hyaluronidase, followed by mechanical removal using a denuding pipette (Vitromed). For ICSI, the oocytes containing the first polar body were positioned at the 12 o'clock location, while the injection needle was positioned at the 3 o'clock location.

### Preparation of PRP

PRP was obtained from fresh blood and peripheral veins. This product was prepared based on the PRP production criteria of the blood transfusion organization. For this purpose, a total of 32 mL of peripheral whole blood was collected from each subject into 4 tubes (8.5 mL BD VacutainerⓇ) containing anticoagulant citrate dextrose solution A. The initial centrifugation (soft spin) was performed at 2500 rpm to separate the supernatant plasma and buffy coat, which were carefully transferred into sterile, empty tubes under aseptic conditions.

A second centrifugation (hard spin) was then carried out to sediment the platelet pellets at the bottom of the tubes. The PRP was subsequently resuspended and transferred into a new sterile tube. This process yielded approximately 5 mL of PRP per sample.

Platelet counts were determined in the baseline whole blood sample using an automated hematology analyzer. The final PRP product was verified to contain a platelet concentration at least threefold higher than the baseline value, in accordance with established PRP preparation standards.

### ET

In this study, the embryos were selected according to the American Society for Reproductive Medicine protocol under abdominal ultrasound and transferred with grade A and B. Embryos were divided into 2 groups: the interventiongroup, which was added PRP to the ET medium (Global) (1:5), and the control group, which used the routine transfer medium for the embryo. To prevent interfering factors, all transfers were performed by one embryologist. The embryos were incubated in a culture medium supplemented with 20% autologous PRP for 3–4 hr prior to transfer. Subsequently, they were transferred into the uterus using the same PRP-enriched medium.

### Examination of chemical and clinical pregnancy

2 wk after ET, a β-hCG test was performed by the clinical laboratory of Fatemiyeh hospital, Hamadan, Iran. A positive test indicated successful implantation. A sonologist defines pregnancy as the presence of embryo's heart rate in the 6 ${}^{\text{th}}$ -12 ${}^{\text{th}}$ wk of pregnancy. Pregnancy was confirmed by an ultrasound with the presence of gestational sac and embryo heart rate by a specialist in radiology. Spontaneous abortion is defined as clinical pregnancy loss before the 20 ${}^{\text{th}}$ wk of pregnancy.

### Ethical Considerations

The study was approved by the Ethics Committee of Hamadan University of Medical Sciences, Hamadan, Iran (Code: IR.UMSHA.REC.1397.235). This study protocol was registered in the clinical trials registry system (ID: IRCT20120215009014N230, updated on November 20, 2024).

### Statistical Analysis

IBM SPSS Statistics version 21 (SPSS 21) software was used for data analysis. The following statistical tests were used for analysis based on the condition: Chi-square test, Fisher's exact test, Student *t *test, Kruskal-Walli's test, and Mann-Whitney test. All analyses were performed at a 95% confidence level. P < 0.05 were considered statistically significant. The results were expressed as mean ± standard deviation (SD).

## 3. Results

**Figure 1:**
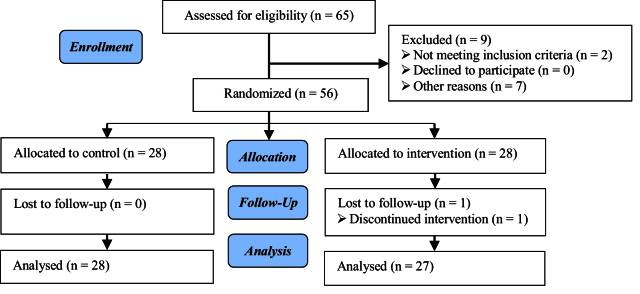
Flowchart of random allocation and follow-up of the study population.

65 individuals who met the initial conditions of the participation and signed the consent form were selected. In total, 9 cases were excluded from the study due to not having the condition of proceeding with treatments or for some other reasons. Ultimately, 56 cases were randomly divided into 2 groups of 28 individuals. One person was excluded from the intervention group due to not following the treatment. Therefore, 55 individuals were included in the study, and the data of 27 cases in the intervention group and 28 cases in the control group were analyzed (Figure 1).

### Primary outcomes

According to the findings of table I, no significant difference was observed between the intervention and control groups in terms of sperm parameters. In the control and intervention groups, the mean age of the women was 28.75 ± 5.46 and 28.67 ± 3.42 yr, respectively, and body mass index (BMI) was 26.24 ± 7.47 and 24.25 ± 3.78 kg/m^2^, and the mean duration of infertility was 4.30 ± 2.49 and 5.71 ± 3.94 yr.

The intervention and control groups were similar in terms of age, BMI, duration of infertility, mean levels of FSH, luteinizing hormone, and estradiol (Table II). In the intervention and control group, in terms of the number of transferred embryos, 2 embryos had the highest frequency, and in terms of embryo grade, grade B had the highest frequency. In terms of the number of transfer embryo cells, 6–8 cell embryos had the highest frequency (Table III). The means ± SD of the transferred embryo in the control and intervention groups were 3.82 ± 2.38 and 4.63 ± 2.34, respectively (p = 0.211).

No statistically significant difference between infertile women in the intervention and control groups in terms of chemical pregnancy or β-hCG titer on the 14 ${}^{\text{th}}$ day after transfer, observation of the pregnancy sac on the day of 21 ${}^{\text{st}}$ after the transfer, and observation of the embryo heart on the 28 ${}^{\text{th}}$ day after transfer (Table IV).

### Secondary outcome

There was one instance of abortion in the control group, while the intervention group did not experience any abortions.

**Table 1:** Analysis of semen parameters of men in different groups

**Variables**	**Control group (n = 28)**		**Intervention group (n = 27)**		**P-value**
	**Number**	**Mean ** ± ** SD**	**Number**	**Mean ± SD**	
Count*	23	41.00 ± 27.00	19	43.36 ± 19.80	0.746
Motility**^a^	19	47.09 ± 32.26 (81.20, 69.20)	19	59.65 ± 35.04 (80.20, 45.85)	0.258
Progressive**^a^	13	32.92 ± 23.69 (35.3, 45)	13	34.85 ± 26.73 (32.00, 54)	0.848
Non-progressive*	13	26.03 ± 17.85	13	38.65 ± 18.69	0.091
Normal morphology*	23	4.17 ± 2.30	19	4.48 ± 4.29	0.763
a: Data presented as Mean ± SD (median, interquartile range). *Student *t* test, **Mann-Whitney test					

**Table 2:** Clinical and paraclinical characteristics of the women

**Variables**	**Control (n = 28)**	**Intervention (n = 27)**	**P-value**
**Age (yr)**	28.75 ± 5.46	28.67 ± 3.42	0.946*
**BMI (kg/m^2^)**	26.24 ± 7.47	24.25 ± 3.78	0.264*
**Duration of infertility (yr)**	4.30 ± 2.49	5.71 ± 3.94	0.264**
**FSH (IU/ml)**	5.43 ± 2.83	7.26 ± 5.84	0.209*
**LH (IU/ml)**	5.59 ± 2.81	6.93 ± 3.65	0.176*
**Estradiol (Pg/ml)**	52.5 ± 25.78	41.08 ± 21.29	0.125*
Data presented as Mean ± SD. *Student *t* test, **Mann-Whitney test. Median (MD) = 4.50 and 4.00; interquartile range (IQR) = 5.50 and 3.37 for control and intervention groups, respectively. BMI: Body mass index, FSH: Follicle-stimulating hormone, LH: Luteinizing hormone			

**Table 3:** Frequency distribution of number and grade of transferred embryos in women in intervention and control groups

**Variables**		**Control group (n = 28)**	**Intervention group (n = 27)**	**P-value**
Number of transferred embryos*				
	**1**	2 (7.2)	3 (11.11)	0.750
	**2**	21 (75)	18 (66.67)	
	**≥ 3**	5 (17.85)	6 (22.22)	
Grade of transferred embryo**				
	**A**	12 (42.86)	12 (44.44)	0.314
	**B**	16 (57.12)	15 (55.56)	
Transferred embryo*				
	**6–8 cells**	23 (82.14)	20 (74)	0.494
	**Morella**	2 (7.2)	4 (14.8)	
	**Blast**	3 (10.66)	5 (18.52)	
Data presented as n (%). *Fisher's exact test, **Chi-square test				

**Table 4:** Frequency distribution of β-hCG results, clinical pregnancy view, and embryo heart view after the transfer, and β-hCG positivity

**Variables**	**Control group (n = 28)**	**Intervention group (n = 27)**	**P-value**
	**Yes**	**Yes**	
**Chemical pregnancy**	11 (39.28)	9 (33.33)	0.646
**Clinical pregnancy**	10 (35.71)	9 (33.33)	0.646
**Embryo heartbeat**	10 (35.71)	9 (33.33)	0.631
**Abortion**	1 (3.57)	0	-
Data presented as n (%). Chi-square test. β-hCG: β-human chorionic gonadotropin			

## 4. Discussion

In the present study, infertile women in the intervention and control groups were similar in terms of age, BMI, duration of infertility, medical history, medication use, pregnancy, and abortion history. Additionally, no significant difference was observed between the 2 groups regarding the mean levels of FSH, luteinizing hormone, and estradiol hormones. In both groups, most transfers involved 2 embryos at the 8-cell stage and grade B. No statistically significant difference was observed between the groups regarding the β-hCG (chemical pregnancy) positivity, presence of a gestational sac, and fetal heart activity.

Successful implantation requires a receptive endometrium, a functional embryo, and coordinated interaction between the blastocyst and the endometrium (17). Poor endometrial receptivity has emerged as a major challenge in infertility treatment, promoting ongoing research into effective therapeutic strategies. Recent studies have shown that intrauterine injection of PRP positively affects reproductive outcomes, including endometrial thickness and clinical pregnancy. Moreover, PRP is considered a less invasive option, as it is derived from the patient's own peripheral blood (18–21). Infertile women with recurrent implantation failure (RIF) may particularly benefit from PRP administration.

One study on women with RIF demonstrated that PRP injection prior to ET could improve the success of implantation (13). Another case reported that intrauterine PRP administration in a 45-yr-old woman with RIF, 24 hr before ET, resulted in a successful implantation and live birth via cesarean section (20).

Similarly, an animal model investigating PRP treatment for thin endometrium showed improved endometrial thickness, with 5 clinical pregnancies out of 10 subjects, following ET in cases with adequate endometrial thickness (11).

In another study involving 20 women aged between 20 and 40 yr with endometrial thickness < 7 mm during a frozen ET cycle, autologous PRP significantly improved endometrial thickness in 71.4% of participants ( ≥ 7 mm). The clinical pregnancy rate was 35%, the implantation rate was 14.2%, and ongoing pregnancy and live birth rates were 14.2% and 20%, respectively. A meta-analysis of 625 women concluded that intrauterine PRP significantly increased endometrial thickness, chemical pregnancy, clinical pregnancy, and implantation compared to controls (21). In a meta-analysis study, the authors concluded that intrauterine injection of autologous PRP in women (n = 625) significantly increased endometrial thickness, chemical pregnancy, clinical pregnancy, and implantation compared to control (22).

However, one study found no statistically significant differences in endometrial thickness and live birth rate between the PRP and control groups, though a significant difference was observed in implantation and clinical pregnancy rates between the groups (12.7 and 30%, respectively) (23).

Another study showed that the endometrium of cases treated with PRP (83 infertile women) increased significantly compared to the control group (without intrauterine injection), and the clinical pregnancy rate was also statistically significant (24).

Hystroscopic PRP instillation at the endomyometrial junction in 32 women with thin endometrium ( < 7 mm) and primary or secondary infertility confirmed endometrial thickness and pregnancy rates (25). Increase in implantation and chemical pregnancy rates in the PRP-treated group were similarly reported by another study (26).

Conversely, another study found no significant difference in pregnancy outcomes including biochemical, clinical, and ongoing pregnancy ( > 20 wk gestation) between RIF participants receiving PRP and those in the control group (27). The mechanisms through which PRP enhances endometrial receptivity have been explored in limited studies. Primarily, PRP promotes cell proliferation, regeneration, and differentiation due to the presence of growth factors and cytokines (22). The PRP stimulates proliferation in various endometrial cell types, including epithelial cells, endometrial stromal fibroblasts, and endometrial mesenchymal stem/progenitor cells (28). PRP treatment has been shown to significantly increase expression of Ki-67, a marker of cell proliferation, in the endometrium (29). PRP may enhance endometrial receptivity by activating the expression of adhesion molecules, recruiting stem cells, and increasing endometrial cell migration (28). It also elevates levels of *Hoxa10*, an early marker of endometrial receptivity (29). Rich in growth factors and cytokines PRP may offer a promising treatment for cases with thin endometrium, particularly those with insufficient vascular endothelial growth factors downregulation. Another possible mechanism involves PRP's anti-inflammatory and antimicrobial properties (30).

PRP increases levels of chemokines such as CCL5, CCL7, CXCL13, and lipoxin A4 while reducing polymorphonuclear neutrophil infiltration, thereby decreasing intrauterine inflammation and fluid accumulation. Furthermore, PRP has been shown to downregulate pro-inflammatory cytokines-including interleukin-1 beta, interleukin-8, tumor necrosis factor-α as well as prostaglandin-endoperoxide synthase 2 and nitric oxide synthase in vitro and in animal models. Antimicrobial peptides in platelet granules may further contribute to these effects (10).

Another potential benefit of PRP is its antifibrotic action. It reduces expression of fibrosis-related factors such as collagen type Iα, transforming growth factor β1, and tissue inhibitor of metalloproteinase-1, thereby promoting tissue remodeling (18). Cytokines in PRP activate matrix metalloproteinases (MMP-1, MMP-3, MMP-7, MMP-26), which are involved in tissue regeneration and wound healing. Thus, PRP may be an effective therapy for intrauterine adhesions by modulating the fibrotic environment (31).

According to the explanation of the mediators in PRP, which undoubtedly had a significant impact on the success of implantation and, ultimately, a successful pregnancy, the addition of these mediators in an exogenous form at the time of ET in the present study may have a direct effect on increasing the receptivity of the endometrium and they do not have successful implantation. Perhaps the mediators in PRP, by inducing and enhancing the uterine factors secreted by the blastocyst, indirectly lead to an increase in the receptivity of the uterus, not with a direct effect on the embryo or decidua of the recipient. Therefore, further elucidation of its mechanism is necessary for its potential clinical application. Also, it would be better to transfer the embryos at the same stage to increase the accuracy of the study.

## 5. Conclusion

In this study, it can be concluded that PRP does not have a direct effect on the embryo and increasing its attachment to the endometrium, but adding it to the uterus increases the fertility rate by improving cell proliferation, angiogenesis, anti-inflammatory properties, and reducing the degree of fibrosis. However, the effect of PRP was not seen in the culture stage and at the time of ET. Despite many studies on the effect of PRP at what stage of implantation and by what mechanism it has led to successful fertilization, it is still unclear. Still, we can be sure of this positive effect during the transfer. However, the best dose and timing of PRP application remain unknown. So, assuming the effectiveness of PRP on other stages of implantation, further investigation on a larger scale is required.

##  Data Availability

Data supporting the findings of this study are available upon reasonable request from the corresponding author.

##  Author Contributions

M. Sanuie Farimani and F. Salari Shahrbabaki, I. Amiri, designed the study. M. Sanuie Farimani, F. Salari Shahrbabaki, A. Moini, A. Aleyasin, and I. Amiri contributed to performing experiments and evaluating the results. Drafting of manuscript for important intellectual content were done by M. Sanuie Farimani, A. Moini, A. Aleyasin, I. Amiri, J. Poorolajal, and statistical analysis was done by J. Poorolajal. All authors approved the final manuscript and take responsibility for the integrity of the data and the accuracy of the data analysis.

##  Conflict of Interest

The authors declare that there is no conﬂict of interest in this study.
